# Single-Center External Validation and Calibration of the ACS NSQIP Surgical Risk Calculator in Emergency Colorectal Cancer Surgery

**DOI:** 10.3390/jcm15218387

**Published:** 2026-10-28

**Authors:** Ahmet Sencer Ergin, Ahmet İlter Ergin, Alparslan Saylar, Ali Karabulut, Nihat Buğdaycı, Serhat Meriç, Hakan Yiğitbaş

**Affiliations:** 1 Department of General Surgery, Bağcılar Training and Research Hospital, 34200 Istanbul, Türkiye; saylaralparslan@gmail.com (A.S.);; 2 Department of General Surgery, Fatih Sultan Mehmet Training and Research Hospital, 34752 İstanbul, Türkiye; 3 Department of General Surgery, Faculty of Medicine, Istanbul Aydın University, 34295 Istanbul, Türkiye

**Keywords:** colorectal cancer, emergency surgery, ACS-NSQIP, surgical risk calculator, external validation, calibration

## Abstract

**Background/Objectives**: Emergency colorectal cancer (CRC) surgery combines acute physiological disturbance with heterogeneous operative decisions. We evaluated the discrimination, calibration, and prediction error of the contemporary American College of Surgeons National Surgical Quality Improvement Program (ACS-NSQIP) Surgical Risk Calculator in an institutional emergency CRC cohort. **Methods**: This retrospective study included 198 patients undergoing clinically indicated emergency CRC resection during 2020–2024. Recorded 30-day outcomes were compared with version 4.0.4 predictions using area under the receiver operating characteristic curve (AUC), observed-to-expected (O/E) ratios, exploratory calibration plots, and Brier scores. Renal-failure analysis included 170 patients, and leak analysis included 118 patients with an anastomosis. **Results**: Mortality occurred in 25 patients (12.6%); discrimination was strong (AUC 0.882, 95% confidence interval [CI] 0.807–0.958), with an O/E ratio of 1.03 (95% CI 0.67–1.52). Pneumonia and cardiac complications had AUCs of 0.768 and 0.799 but were underpredicted (O/E 3.48 and 3.62). Surgical site infection occurred in 40.4%, with limited discrimination (AUC 0.544, 95% CI 0.462–0.626) and underprediction (O/E 3.15, 95% CI 2.49–3.92). Anastomotic leak occurred in 12 of 118 patients (10.2%). Mean absolute error for hospital stay was 5.46 days. **Conclusions**: Mortality ranking was strong, while absolute-risk estimation differed by outcome. Larger multicenter validation with consistent outcome definitions is necessary before these probabilities can support reliable individual risk communication in emergency CRC surgery.

## 1. Introduction

Colorectal cancer (CRC) remains a major cause of cancer morbidity and mortality worldwide [1]. A clinically important subgroup presents with obstruction, perforation, or bleeding requiring emergency surgical assessment. Earlier series estimate that approximately 15–30% of CRC presents as an emergency, although the proportion depends on the population and definition used [2]. Emergency presentation has been associated with adverse short-term outcomes and, in several cohorts, poorer longer-term survival [3,4,5,6]. These associations reflect a combination of acute illness, underlying health, tumor characteristics, and treatment circumstances; emergency presentation alone does not explain every difference in prognosis [7].

Risk estimation in this setting is difficult because the clinical state may evolve during resuscitation and surgery. Obstruction can coexist with dehydration, organ dysfunction, or advanced malignancy, whereas perforation adds contamination and systemic inflammation. Time for nutritional assessment, optimization of comorbidities, and detailed counseling may be limited. Patients undergoing emergency CRC surgery therefore differ from the broader surgical population in ways beyond their emergency designation. A binary emergency variable and a principal procedure code cannot describe every aspect of physiological deterioration or operative complexity [8].

The operation itself is also heterogeneous. The choice between resection with anastomosis, diversion, and an end stoma depends on the patient’s condition and intraoperative findings. In a prospective multicenter emergency colorectal resection cohort, patients selected for a stoma had a different preoperative and operative risk profile from those undergoing anastomosis [9]. Such selection complicates interpretation of observed complication rates: an association between procedure type and an adverse outcome may reflect the reasons for selecting that procedure. An emergency CRC validation cohort must therefore describe its operative mix and distinguish outcomes that apply to all patients from those requiring an anastomosis.

The ACS-NSQIP Surgical Risk Calculator combines patient characteristics with the planned Current Procedural Terminology (CPT) code to estimate postoperative risks. It was developed as a decision aid to support communication and informed consent using data from multiple institutions [10]. Its current implementation provides estimates for specified 30-day outcomes and hospital stay, with some procedure-specific outcomes displayed conditionally [11,12]. This broad coverage makes the calculator attractive in emergency practice, where a standardized estimate can complement an otherwise time-limited assessment. However, predictions derived in one clinical setting require evaluation in the population in which they will be used.

Previous colorectal and emergency-surgery validations have produced heterogeneous findings [13,14,15,16,17,18,19,20]. Scotton et al. reported favorable performance in an Italian multicenter emergency abdominal surgery cohort [19], whereas van der Hulst et al. found poor calibration for several outcomes in older patients undergoing elective CRC surgery [14]. Other colorectal studies have highlighted the distinction between acceptable prediction of a cohort’s overall event burden and accurate probabilities for individual patients [15,16,17,18]. These studies differ in case mix, outcome definitions, sample size, and calculator generation. Contemporary machine-learning results from ACS-NSQIP itself also indicate that high operative risk does not necessarily imply poor model performance [21].

Discrimination and calibration answer different clinical questions. Discrimination assesses whether higher-risk patients tend to experience more events; calibration assesses whether estimated probabilities match observed frequencies. A model can discriminate reasonably well while systematically underestimating absolute risk, and agreement in an overall event rate can conceal discrepancies across risk levels [22,23,24]. We therefore evaluated outcome-specific discrimination, aggregate observed-to-expected agreement, grouped calibration, and probabilistic error of the contemporary ACS-NSQIP calculator in patients undergoing emergency CRC resection at our institution. We also examined predicted versus observed hospital stay and the compatibility of recorded endpoints with the calculator targets.

## 2. Materials and Methods

### 2.1. Study Design and Cohort Selection

**Figure 1:**
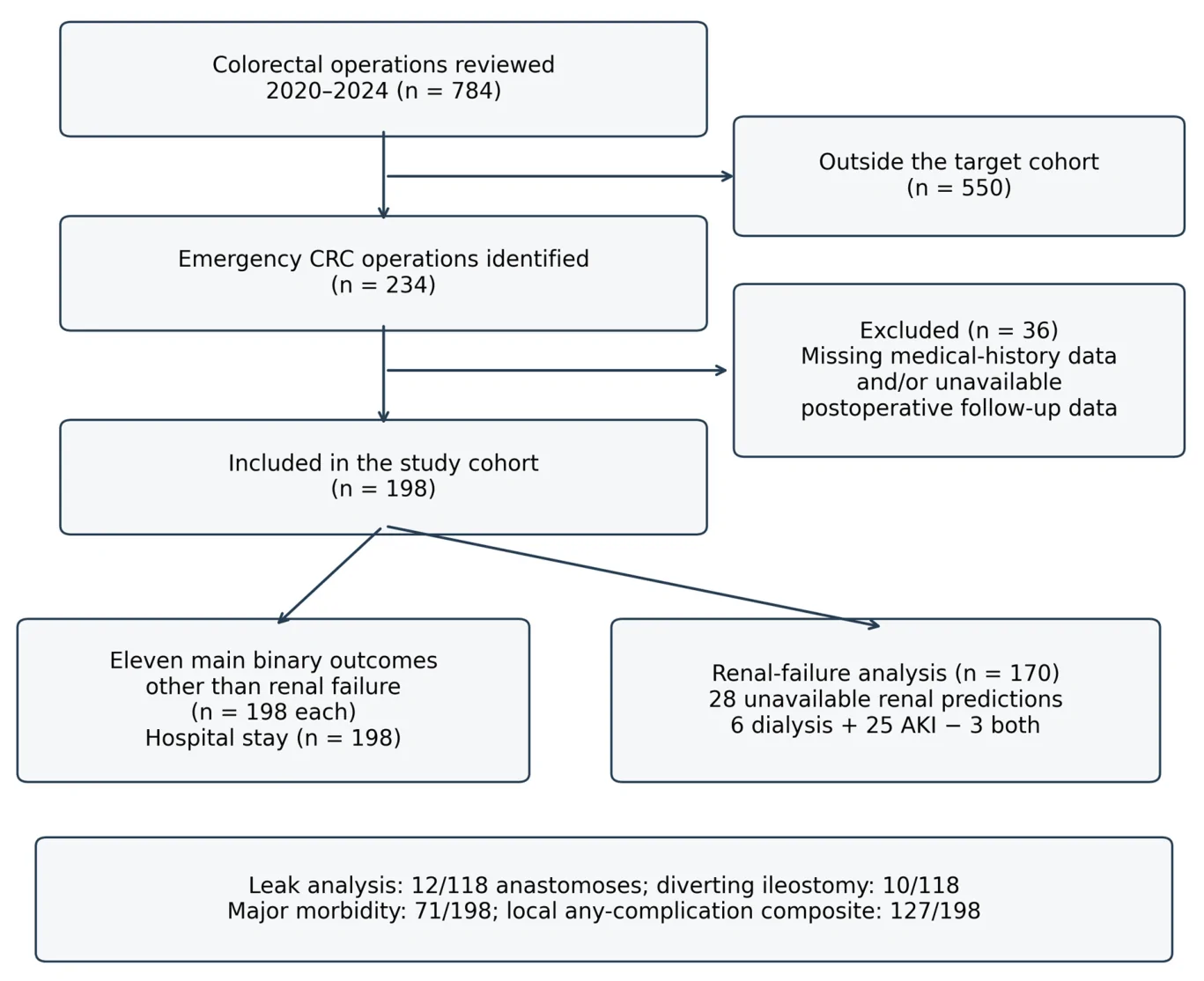
Cohort selection and outcome-specific analysis sets. Thirty-six patients were excluded because of insufficient medical-history information for ACS-NSQIP risk estimation and/or unavailable postoperative follow-up records for 30-day outcome ascertainment. These exclusions are reported as one combined data-insufficiency category. AKI—stage 2/3 acute kidney injury; CRC—colorectal cancer.

This retrospective study reviewed 784 colorectal operations performed at Bağcılar Training and Research Hospital during 2020–2024. The study population comprised patients identified in the institutional dataset as undergoing emergency CRC resection. Elective operations, cytoreductive surgery, non-malignant indications, and ostomy without tumor resection were outside the target cohort. Of 234 emergency CRC operations identified during screening, 36 patients were excluded because the hospital information system lacked medical-history information required for ACS-NSQIP risk estimation and/or postoperative follow-up data were unavailable for 30-day outcome ascertainment. These 36 exclusions were grouped as insufficient data for risk estimation and/or postoperative outcome ascertainment, leaving 198 patients for analysis (Figure 1).

The treating surgical team defined emergency surgery by documented assessment of clinical urgency and the need for an unplanned operation. Indications included acute abdominal findings, persistent mechanical bowel obstruction that did not resolve during observation and conservative treatment, including observation for up to 72 h, and clinical deterioration associated with mechanical obstruction requiring urgent decompression. Eligibility was based on clinical urgency rather than a fixed interval from admission to surgery. Elective procedures were excluded. The external-validation designation refers to assessment in an institutional cohort separate from the model-development data; it does not imply multicenter sampling or establish transportability to other hospitals [23].

The Institutional Review Board of Bağcılar Training and Research Hospital approved the study on 10 April 2026 (approval No. 2026/04/02/029). Retrospective medical-record review commenced after ethics committee approval. Written informed consent for the research use of clinical data was obtained from patients or their next of kin, as applicable. The study was conducted in accordance with the Declaration of Helsinki.

### 2.2. Data Preparation and Risk Estimation

Preoperative characteristics, operative procedures, and postoperative outcomes were abstracted from the medical records and linked to the corresponding calculator predictions using unique study identifiers. The dataset contained 198 distinct patients. Height and weight were recorded in centimeters and kilograms, respectively, and body mass index was calculated as weight divided by squared height in meters.

Calculator inputs comprised age, sex, body mass index, functional status, emergency status, ASA class, immunosuppressive therapy, ascites, preoperative systemic sepsis, ventilator dependence, disseminated cancer, diabetes, treated hypertension, congestive heart failure, oxygen support, smoking, severe chronic obstructive pulmonary disease, dialysis, stage 2/3 acute kidney injury, and the procedure code. Wound class and recorded TNM information were used to describe the cohort. TNM stage was not entered as a calculator predictor. Recorded CPT codes and their procedural meanings are summarized in Table 1. The operative distribution included 118 anastomoses, including 10 with a diverting ileostomy.

All patient characteristics and procedure codes were entered individually into the ACS-NSQIP Surgical Risk Calculator version 4.0.4 (last parameter update: April 2026) [11], using the default surgeon-adjustment setting without modification. The emergency-surgery field was set to “Yes” for every patient. Predicted probabilities were recorded for comparison with observed outcomes. The calculator was applied retrospectively to operations performed during 2020–2024; this study therefore evaluated retrospective predictive performance rather than predictions used for contemporaneous clinical decisions.

No numerical imputation of calculator inputs or outcome probabilities was performed. Patients with insufficient information for risk estimation or postoperative outcome ascertainment were excluded during cohort selection. Diabetes was categorized as absent, treated with oral agents, or treated with insulin; patients without diabetes were entered as “No diabetes” in the calculator.

### 2.3. Outcomes and Endpoint Compatibility

Outcomes were assessed during the first 30 days after surgery. Postoperative pneumonia, cardiac complications, venous thromboembolism, sepsis, SSI, urinary tract infection, renal failure, and ileus were identified from diagnoses documented by the treating clinical team in the medical records. These diagnoses reflected routine clinical assessment, supported by vital signs, laboratory investigations, including biochemical parameters, and chest radiographs, as clinically indicated. Recorded diagnoses were used for outcome classification without additional retrospective adjudication against prespecified standardized diagnostic criteria. Events after discharge were ascertained from emergency department, outpatient clinic, and readmission records within the same postoperative 30-day period.

All readmissions were unplanned, and all returns to the operating room were unplanned emergency procedures; planned events were excluded from these endpoints. Postoperative hospital stay was measured in days from surgery to discharge. Each binary outcome was compared with its corresponding calculator probability. The ACS targets include cardiac arrest or myocardial infarction, venous thrombosis or pulmonary embolism, and progressive renal insufficiency or acute renal failure [12]. These target descriptions were distinguished from the routine clinical diagnoses used in this study, for which strict concordance with ACS diagnostic criteria was not established. SSI was evaluated as a single binary endpoint. Supplementary Table S3 specifies the recorded variables, outcome ascertainment, and corresponding calculator targets.

Major morbidity was defined as Clavien–Dindo grade III or higher [25]. This severity-based endpoint differs from the ACS-NSQIP serious-complication composite, which combines specified adverse events [12]. Major morbidity was reported descriptively and was not compared directly with the calculator’s serious-complication probability.

Anastomotic leak was distinguished from stoma or mucous-fistula dehiscence and complications of a closed distal stump. Leak analysis included the 118 patients with an anastomosis (CPT codes 44140, 44146, and 44160), including 10 patients with a diverting ileostomy. Patients without an anastomosis, including Hartmann procedures and the patient who underwent total abdominal colectomy with an end ileostomy (CPT 44150), were excluded from the leak-analysis numerator and denominator. The five patients coded 44146 underwent anastomosis with a diverting ileostomy. No comparison of leak risk by diversion status was undertaken.

The local “any complication” composite was positive when at least one recorded postoperative complication was present, including ileus. Readmission or discharge destination alone was not treated as proof of a complication. This local definition is broader than the ACS-NSQIP any-complication target, which lists specified adverse events and treats ileus as a separate procedure-specific outcome [12]. Its comparison with the calculator’s any-complication probability was therefore a secondary exploratory analysis.

### 2.4. Outcome-Specific Analysis Sets

Patients without a numerical outcome-specific prediction were excluded only from that outcome analysis. Renal-failure probabilities were recorded as “none” in 28 patients with preoperative renal dysfunction: 6 had dialysis, 25 had stage 2/3 acute kidney injury, and 3 had both. These entries represented unavailable calculator outputs, rather than zero risk or imputed missing input values. Renal-failure analysis included 170 patients, leak analysis 118 patients with an anastomosis, and each remaining binary outcome 198 patients. The analysis script checks the relevant numerator and denominator for every outcome.

### 2.5. Statistical Analysis and Reproducibility

Continuous characteristics were summarized as medians and interquartile ranges (IQR), and categorical characteristics as counts and percentages. Baseline characteristics were presented descriptively for the entire cohort. No new model was developed, predictors were not selected, and the original predictions were not recalibrated to this dataset.

Discrimination was quantified by AUC with a 95% CI based on DeLong variance [26]. Expected event counts were calculated as the sum of individual predicted probabilities in the same patients contributing observed events. O/E ratios were calculated by dividing observed events by expected events; exact Poisson 95% CIs were calculated with the expected total treated as fixed. An O/E ratio above 1 indicates more observed than predicted events. Its uncertainty was considered when interpreting apparent aggregate agreement.

Exploratory calibration plots compared observed proportions with mean predicted risks in up to ten quantile-based groups. Tied predictions were retained within the same group, so group sizes could differ. Every displayed group includes its patient and event counts, and observed proportions have exact binomial 95% CIs. Full group-level results are provided in Supplementary Table S2. The Hosmer–Lemeshow statistic used these groups and degrees of freedom equal to the number of groups minus two. It was supplementary: a non-significant result was not interpreted as evidence that individual probabilities were well calibrated, particularly with few events [24]. Calibration intercepts and slopes were not estimated.

Brier scores were calculated as the mean squared difference between predicted probabilities and observed binary outcomes. Their 95% percentile bootstrap CIs used 2000 resamples of patient records. Brier scores were interpreted as outcome-specific prediction errors because outcome frequency affects their magnitude. Hospital-stay agreement was assessed with observed and predicted medians, mean signed error (observed minus predicted), median signed error, mean absolute error, and root mean squared error. Positive signed error indicates underprediction of stay. Bootstrap CIs for mean signed and absolute errors used 2000 resamples; the fixed random seed was 20260929.

The available eligible cohort determined sample size; no a priori precision-based calculation was performed. Rare-event and grouped calibration results were treated as exploratory, and no multiplicity-adjusted confirmatory claims were made. Analyses used Python 3.12.14, NumPy 2.3.5, pandas 2.2.3, SciPy 1.17.0, scikit-learn 1.8.0, and Matplotlib 3.10.8. Supplementary Code S1 contains the analysis script, software requirements, calculation details, and aggregate output tables. OpenAI ChatGPT-6 Astra assisted with analysis-code development and execution and data-consistency checks. Following AI-assisted analysis, all statistical methods, outputs, and reported results were reviewed and verified by the authors. The authors take full responsibility for the accuracy and integrity of the analyses and their interpretation.

## 3. Results

### 3.1. Cohort and Operative Characteristics

The cohort included 198 patients, with a median age of 67.0 years (IQR 55.2–74.0); 73 patients (36.9%) were women. ASA class III–IV was recorded in 117 patients (59.1%), and 113 (57.1%) were partially or totally dependent. Disseminated malignancy was recorded in 55 patients (27.8%). Diabetes was absent in 111 patients (56.1%); 66 (33.3%) used oral therapy, and 21 (10.6%) used insulin. Table 2 provides the remaining baseline characteristics; two nonstandard nodal-stage entries remain unclassified.

The most frequent procedure codes were 44143 and 44160; each was recorded in 64 patients (32.3%). The other codes were 44140 in 49 (24.7%), 44144 in 15 (7.6%), 44146 in 5 (2.5%), and 44150 in 1 (0.5%) (Table 1). Contaminated or dirty/infected wounds were recorded in 97 patients (49.0%). Anastomosis was performed in 118 patients (59.6%), including 10 with a diverting ileostomy (8.5% of anastomoses). Clavien–Dindo grade III or higher morbidity occurred in 71 patients (35.9%). The local any-complication composite occurred in 127 patients (64.1%).

### 3.2. Observed and Expected Outcomes

Thirty-day mortality occurred in 25 patients (12.6%), compared with 24.3 expected deaths (12.2%). The mortality O/E ratio was 1.03 (95% CI 0.67–1.52), indicating similar aggregate totals but appreciable uncertainty. Pneumonia occurred in 50 patients (25.3%) compared with 14.4 expected events (7.3%; O/E 3.48, 95% CI 2.58–4.58). Cardiac complications occurred in 28 patients (14.1%) compared with 7.7 expected events (3.9%; O/E 3.62, 95% CI 2.41–5.23).

SSI was recorded in 80 patients (40.4%) compared with 25.4 expected events (12.8%; O/E 3.15, 95% CI 2.49–3.92). Sepsis and urinary tract infection were also underpredicted, with O/E ratios of 2.25 (95% CI 1.70–2.93) and 2.40 (95% CI 1.15–4.41), respectively. In contrast, discharge to a nursing or rehabilitation facility was recorded in 4 patients (2.0%) compared with 55.7 expected events (28.1%; O/E 0.07, 95% CI 0.02–0.18).

Within the renal-failure analysis set, 13 of 170 patients (7.6%) had a recorded event, compared with 10.0 expected events (5.9%; O/E 1.29, 95% CI 0.69–2.21). O/E ratios for venous thromboembolism and ileus were 0.91 (95% CI 0.37–1.88) and 0.93 (95% CI 0.72–1.18). Their CIs do not establish equivalence between observed and predicted risks. Among 118 patients with an anastomosis, 12 leaks occurred (10.2%), compared with 5.6 expected events (4.8%; O/E 2.13, 95% CI 1.10–3.72). Table 3 reports the main outcome-specific observed and expected counts.

### 3.3. Discrimination and Calibration

**Figure 2:**
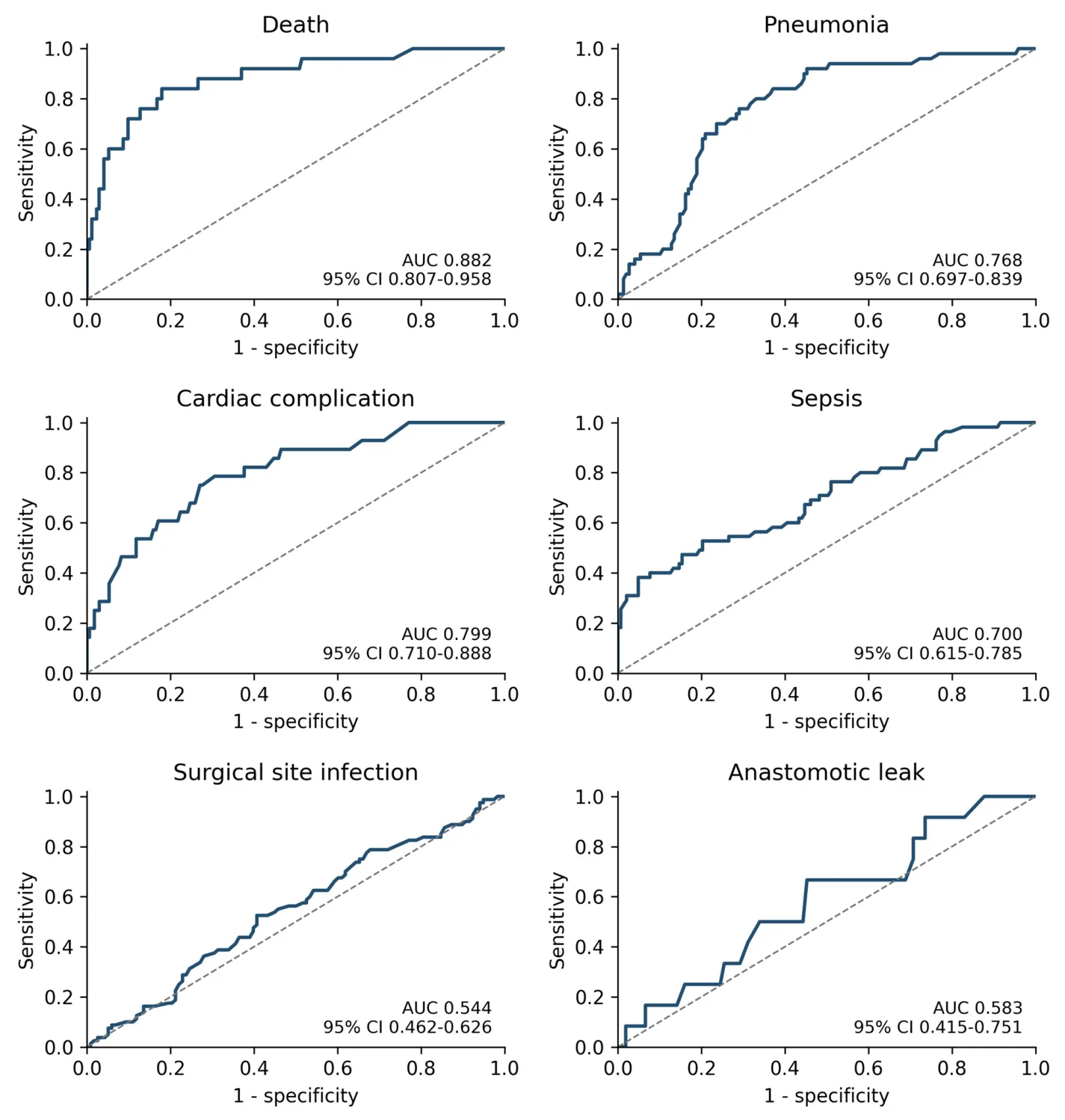
Receiver operating characteristic curves for six selected recorded outcomes. AUC and DeLong 95% CI are shown in each panel. Anastomotic leak includes 118 patients; each other displayed outcome includes 198 patients. The local any-complication composite is reported separately as a secondary comparison.

Discrimination was strongest for mortality (AUC 0.882, 95% CI 0.807–0.958), followed by cardiac complications (0.799, 95% CI 0.710–0.888) and pneumonia (0.768, 95% CI 0.697–0.839). SSI discrimination was limited (0.544, 95% CI 0.462–0.626), as was discrimination for readmission (0.515, 95% CI 0.403–0.626) and return to the operating room (0.537, 95% CI 0.427–0.648). Leak AUC in the anastomosis subset was 0.583 (95% CI 0.415–0.751). Estimates for venous thromboembolism and discharge to a care facility were particularly imprecise because there were only seven and four events. AUC and Brier-score CIs are reported in Table 4; selected ROC curves are shown in Figure 2.

**Figure 3:**
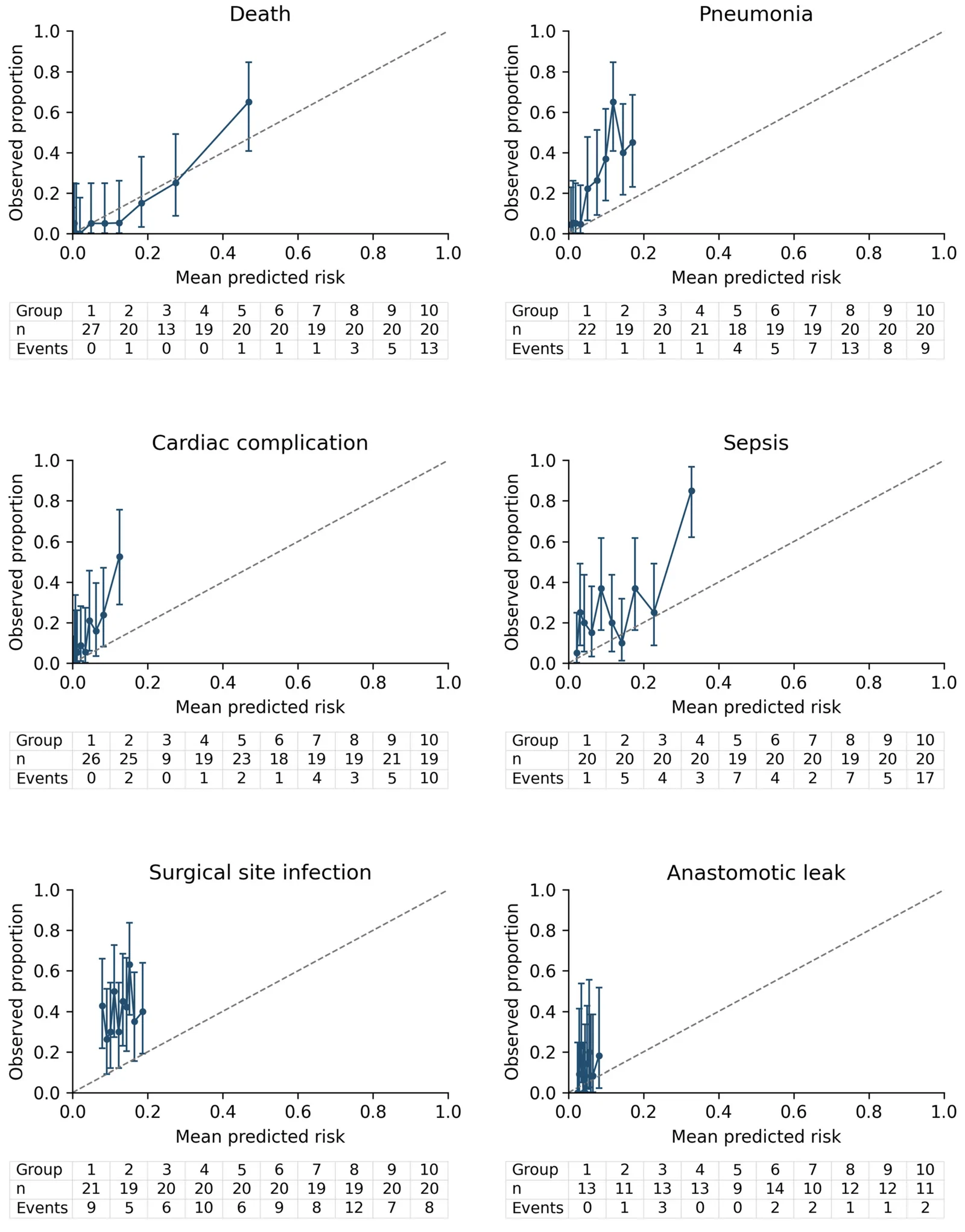
Exploratory grouped calibration for six selected outcomes. Points show observed event proportions at group mean predicted risks; vertical bars are exact binomial 95% CIs. The dashed line represents perfect agreement. The tables below each panel give group number, patient count (n), and event count. Groups are based on prediction quantiles, with ties retained together. Small event counts limit precision; these plots do not establish individual-level calibration.

The grouped plots illustrate outcome-specific differences between observed and predicted risks and substantial uncertainty in groups with few events (Figure 3). Mortality had a non-significant supplementary Hosmer–Lemeshow result (χ^2^ = 14.00; *p* = 0.082), which did not support good calibration. The apparent aggregate mortality agreement should be interpreted together with the O/E CI and group-level uncertainty. Pneumonia, cardiac complications, sepsis, and SSI showed marked underprediction in aggregate. Full numerical calibration results are provided in the Supplementary Tables.

### 3.4. Hospital Length of Stay

Observed postoperative hospital stay was a median of 7.0 days (IQR 6.0–11.0), compared with a predicted median of 8.5 days (IQR 6.5–9.5). The median paired difference was 0 days, but the mean signed error was +2.46 days (95% CI 1.05–4.09), reflecting longer observed stays on average. Mean absolute error was 5.46 days (95% CI 4.21–6.86), and root mean squared error was 10.97 days (Table 5). Similar central values therefore did not imply close patient-level agreement.

### 3.5. Secondary Comparison of the Local Complication Composite

The local composite occurred in 127 patients (64.1%), compared with 62.4 expected events (31.5%) from the calculator’s any-complication output. Its AUC was 0.709 (95% CI 0.636–0.782), and its O/E ratio was 2.04 (95% CI 1.70–2.42). Because the local definition includes ileus, these estimates combine model performance with an endpoint-definition difference and should not be interpreted as direct validation of the ACS composite. The complete secondary results are provided in Supplementary Table S1.

## 4. Discussion

This institutional evaluation found strong discrimination for 30-day mortality and similar aggregate observed and predicted mortality totals. Performance differed across other endpoints. Pneumonia and cardiac complications retained useful ranking performance while their absolute risks were substantially underpredicted. SSI combined limited discrimination with substantial underprediction. These patterns have different implications: improving average probability estimates alone would not resolve weak SSI ranking, whereas an outcome with reasonable discrimination may still require correction of systematic probability error before use in individual counseling.

The mortality findings warrant a measured interpretation. An AUC of 0.882 indicates that the calculator usually assigned higher risks to patients who died than to survivors. The mortality O/E point estimate was close to 1, but its CI ranged from 0.67 to 1.52, and calibration groups contained few deaths. These results support useful mortality ranking in this cohort without establishing precise individual probabilities. Similarly, the non-significant Hosmer–Lemeshow test is compatible with limited power and depends on the grouping scheme. Its *p*-value cannot establish good calibration [24].

Our findings fit a broader literature in which performance varies across colorectal and emergency-surgery cohorts [13,14,15,16,17,18,19,20]. The favorable results reported by Scotton et al. in emergency abdominal surgery and the poor calibration observed by van der Hulst et al. in older elective CRC patients show why findings cannot be transferred between populations on the basis of surgical specialty alone [14,19]. Differences in emergency definitions, operative selection, endpoints, and calculator versions may all contribute. Contemporary ACS analyses have reported favorable performance across multiple high-risk subsets, including emergency and high-ASA populations [21]. Our data therefore identify possible local prediction discrepancies; they do not demonstrate that high-risk emergency patients inherently fall outside the calculator’s scope.

Operative context is a plausible contributor to local differences. Almost half of this cohort had contaminated or dirty/infected wounds, and 64 operations were coded as Hartmann procedures. Bowel distension, ischemia, contamination, tissue viability, and the need for abbreviated or open-abdomen management may alter reconstruction and subsequent risk. A preoperative estimate cannot fully incorporate findings that become apparent only during surgery. These mechanisms were not tested as independent explanations in this study. Observational comparisons of anastomosis and stoma also require attention to selection by clinical severity, as shown in multicenter emergency colorectal research [9]. Of 118 patients with an anastomosis, 10 received a diverting ileostomy; these aggregate counts describe operative selection but do not establish the effect of diversion on leak risk.

Leak assessment requires an eligible denominator. Stoma dehiscence and distal-stump complications were excluded from the leak endpoint, and the analysis was restricted to the 118 patients with an anastomosis. The observed incidence was 10.2%, with only 12 events available to assess discrimination and calibration. This limited event count constrains precision and precludes reliable inference about the protective effect of diversion. Composite endpoints also require consistent definitions. Clavien–Dindo grade III or higher morbidity was retained as a descriptive severity measure, while the local any-complication composite was analyzed secondarily because its inclusion of ileus makes it broader than the ACS target.

SSI deserves particular attention because it can require wound treatment, prolong recovery, increase healthcare contacts, and add costs. Costeira et al. found higher hospital costs after incisional SSI in a matched colorectal cohort, with greater hospitalization and outpatient resource use [27]. In our cohort, SSI affected 40.4% of patients and had an AUC close to 0.5. The large O/E difference may reflect differences in local exposure, ascertainment, or definition, as well as model calibration. Treatment consequences and attributable costs were not quantified in our dataset.

Additional biomarkers may eventually complement clinical prediction, but their timing and incremental value require explicit evaluation. Verras and Mulita associated lower early postoperative butyrylcholinesterase levels with SSI in a prospective colorectal cohort [28]. This is relevant to postoperative surveillance, but it does not establish that the marker improves preoperative ACS-NSQIP predictions or performs similarly in emergency CRC. Butyrylcholinesterase was not measured in our study, and its added value cannot be inferred from the present results.

Potential applications of risk estimates include informed consent and planning postoperative observation. During a time-limited emergency consent discussion, an estimate may help structure discussion of death and morbidity, but present numerical probabilities with their uncertainty and interpret them in the context of the anticipated operation. For postoperative triage, the initial estimate should be reconsidered alongside the patient’s evolving physiology and operative findings. Our results do not define thresholds for ICU admission or reduced ward surveillance. Predictions were evaluated retrospectively, and we did not measure whether their clinical use changed monitoring, resource allocation, decision quality, or outcomes.

Hospital-stay results reinforce the difference between aggregate description and individual prediction. The mean absolute error exceeded five days despite relatively similar median observed and predicted stays. A small median signed difference can coexist with important error for individual patients and a long tail of prolonged admissions. Hospital stay and discharge destination also depend on local care pathways and available post-acute facilities, while early death can shorten observed stay. The marked overprediction of discharge to a nursing or rehabilitation facility should therefore be interpreted in the institutional context rather than as an isolated measure of model quality.

Several limitations affect interpretation. The retrospective, single-center design permits selection and ascertainment bias and limits generalizability. Complications were identified from routine clinical diagnoses rather than retrospectively adjudicated against standardized ACS-NSQIP criteria. Differences in outcome definitions and ascertainment may therefore have influenced event rates and estimates of calculator performance. Review of emergency department, outpatient, and readmission records cannot exclude events managed outside the available record system. Emergency eligibility reflected clinical urgency rather than a fixed admission-to-surgery interval, which should be considered when comparing cohorts. Exclusion of 36 patients because of insufficient medical-history information and/or unavailable postoperative follow-up records may introduce selection bias. Only 12 anastomotic leaks occurred, limiting precision and preventing reliable assessment of the effect of diversion. Retrospective procedure codes also do not indicate which operation was planned before incision. The local broad complication composite differs from the ACS target, so its comparative estimates remain exploratory. The cohort was modest for external validation, particularly for uncommon outcomes; required sample size depends on event prevalence, risk distribution, and the precision sought [29]. Single-institution discrepancies can also reflect local pathways and data quality [30]. We did not estimate calibration slopes or intercepts, perform recalibration or decision-curve analysis, or evaluate clinical implementation.

Future work should harmonize outcome definitions and prospectively document operative reconstruction, urgency, and complete 30-day follow-up across hospitals. Multicenter validation with an adequate event count could then determine whether the direction and magnitude of the observed discrepancies are reproducible. If systematic miscalibration persists, recalibration should be developed and evaluated in separate data before clinical implementation. A dedicated emergency CRC model should not be assumed to outperform a universal model, particularly if developed in a much smaller sample [31]. Subsequent implementation studies should test whether risk-informed counseling and triage improve decisions or outcomes, rather than inferring clinical benefit from AUC alone.

## 5. Conclusions

The contemporary ACS-NSQIP calculator showed strong mortality discrimination and similar aggregate observed and predicted mortality in this emergency CRC cohort. Several nonfatal outcomes were underpredicted, and SSI also showed limited discrimination. Leak incidence was 10.2% among patients with an anastomosis. These results do not establish accurate individual probabilities or the benefit of calculator-guided care. Larger multicenter validation with consistent endpoint definitions is needed before using these estimates to guide individual risk communication in this setting.

## Figures and Tables

**Table 1:** Recorded CPT codes and their procedural meanings.

CPT	Procedural Meaning of the Recorded Code	n (%)
44140	Partial colectomy with anastomosis	49 (24.7)
44143	Partial colectomy with an end colostomy and closed distal segment (Hartmann type)	64 (32.3)
44144	Partial colectomy with a colostomy or ileostomy and mucous fistula	15 (7.6)
44146	Partial colectomy with a low pelvic colorectal anastomosis and colostomy	5 (2.5)
44150	Total abdominal colectomy without proctectomy, with ileostomy or ileorectal anastomosis	1 (0.5)
44160	Partial colectomy with terminal ileal resection and ileocolic anastomosis	64 (32.3)

Descriptions summarize CPT code meanings. In this cohort, the five patients who were coded 44146 had a diverting ileostomy, and the patient coded as 44150 had an end ileostomy without an anastomosis. Overall, 118 patients had an anastomosis (59.6% of the cohort), including 10 with a diverting ileostomy (8.5% of anastomoses). Leak analysis included the patients coded 44140, 44146, and 44160. No subgroup comparison by diversion status was performed.

**Table 2:** Baseline characteristics of the 198 included patients.

Characteristic	n (%) or Median (IQR)
Age, years	67.0 (55.2–74.0)
Female sex	73 (36.9)
Body mass index, kg/m^2^	26.1 (23.9–29.0)
Disseminated malignancy	55 (27.8)
Functional status: Independent	85 (42.9)
Functional status: Partially dependent	66 (33.3)
Functional status: Totally dependent	47 (23.7)
ASA class 1	19 (9.6)
ASA class 2	62 (31.3)
ASA class 3	100 (50.5)
ASA class 4	17 (8.6)
Immunosuppressive therapy	12 (6.1)
Ascites within 30 days	62 (31.3)
Ventilator dependence	14 (7.1)
Hypertension requiring medication	77 (38.9)
Congestive heart failure within 30 days	61 (30.8)
Oxygen support	43 (21.7)
Current smoking within 1 year	53 (26.8)
Severe COPD	37 (18.7)
Preoperative dialysis	6 (3.0)
Stage 2/3 acute kidney injury	25 (12.6)
Preoperative sepsis: None	127 (64.1)
Preoperative sepsis: SIRS	65 (32.8)
Preoperative sepsis: Sepsis	6 (3.0)
Preoperative sepsis: Septic shock	0 (0.0)
Diabetes: None recorded	111 (56.1)
Diabetes: Oral therapy	66 (33.3)
Diabetes: Insulin	21 (10.6)
Wound class: Clean-contaminated	101 (51.0)
Wound class: Contaminated	89 (44.9)
Wound class: Dirty/infected	8 (4.0)
T4 disease	131 (66.2)
Node-positive disease	139 (70.2)
Nodal stage not classifiable	2 (1.0)

Percentages use n = 198. Continuous values use the 25th and 75th percentiles. Nodal stage was unclassifiable for two patients. ASA—American Society of Anesthesiologists; COPD—chronic obstructive pulmonary disease; SIRS—systemic inflammatory response syndrome. The table describes the full cohort.

**Table 3:** Observed and expected 30-day outcomes in the same eligible patients.

Outcome	n	Observed n (%)	Expected n (%)	O/E Ratio (95% CI)
Pneumonia	198	50 (25.3)	14.4 (7.3)	3.48 (2.58–4.58)
Cardiac complication	198	28 (14.1)	7.7 (3.9)	3.62 (2.41–5.23)
Venous thromboembolism	198	7 (3.5)	7.7 (3.9)	0.91 (0.37–1.88)
Sepsis	198	55 (27.8)	24.4 (12.3)	2.25 (1.70–2.93)
Surgical site infection	198	80 (40.4)	25.4 (12.8)	3.15 (2.49–3.92)
Urinary tract infection	198	10 (5.1)	4.2 (2.1)	2.40 (1.15–4.41)
Renal failure	170	13 (7.6)	10.0 (5.9)	1.29 (0.69–2.21)
Unplanned readmission	198	34 (17.2)	24.8 (12.5)	1.37 (0.95–1.91)
Unplanned return to operating room	198	27 (13.6)	18.3 (9.2)	1.48 (0.97–2.15)
Death	198	25 (12.6)	24.3 (12.2)	1.03 (0.67–1.52)
Nursing/rehabilitation discharge	198	4 (2.0)	55.7 (28.1)	0.07 (0.02–0.18)
Ileus	198	67 (33.8)	72.3 (36.5)	0.93 (0.72–1.18)
Anastomotic leak	118	12 (10.2)	5.6 (4.8)	2.13 (1.10–3.72)

Expected counts are the sum of individual probabilities; their percentages are the mean predicted risk. O/E confidence intervals use an exact Poisson method, treating the expected count as fixed. Renal failure includes 170 patients after exclusion of 28 unavailable predictions. Anastomotic leak includes 118 patients with an anastomosis; the remaining outcomes include 198 patients. Clavien–Dindo major morbidity is descriptive only. The local any-complication composite is analyzed secondarily in Supplementary Table S1 because its inclusion of ileus makes it broader than the ACS target. Outcomes were ascertained from clinical records within 30 days after surgery. Readmissions and returns to the operating room were unplanned.

**Table 4:** Discrimination and probabilistic prediction error.

Outcome	AUC (95% CI)	Brier Score (95% CI)
Pneumonia	0.768 (0.697–0.839)	0.204 (0.157–0.253)
Cardiac complication	0.799 (0.710–0.888)	0.121 (0.083–0.163)
Venous thromboembolism	0.602 (0.353–0.851)	0.034 (0.011–0.058)
Sepsis	0.700 (0.615–0.785)	0.202 (0.158–0.248)
Surgical site infection	0.544 (0.462–0.626)	0.316 (0.265–0.371)
Urinary tract infection	0.575 (0.387–0.763)	0.049 (0.020–0.082)
Renal failure	0.683 (0.506–0.859)	0.067 (0.036–0.101)
Unplanned readmission	0.515 (0.403–0.626)	0.146 (0.108–0.186)
Unplanned return to operating room	0.537 (0.427–0.648)	0.120 (0.081–0.159)
Death	0.882 (0.807–0.958)	0.076 (0.053–0.101)
Nursing/rehabilitation discharge	0.643 (0.376–0.910)	0.141 (0.118–0.163)
Ileus	0.584 (0.499–0.669)	0.227 (0.204–0.250)
Anastomotic leak	0.583 (0.415–0.751)	0.094 (0.048–0.145)

AUC CIs use DeLong variance. Brier CIs are percentile intervals from 2000 patient-record bootstrap resamples. Brier scores are prevalence-dependent and should not be ranked across different outcomes as if they shared a common baseline. Analysis sets are the same as in Table 3. AUC, area under the receiver operating characteristic curve; CI—confidence interval.

**Table 5:** Observed and predicted postoperative hospital stay.

Measure	Value
Observed stay, median (IQR), days	7.0 (6.0–11.0)
Predicted stay, median (IQR), days	8.5 (6.5–9.5)
Mean signed error, days (95% CI)	2.46 (1.05–4.09)
Median signed error, days	0.00
Mean absolute error, days (95% CI)	5.46 (4.21–6.86)
Root mean squared error, days	10.97

All 198 patients contributed paired observations. Observed stay was measured from surgery to discharge. Signed error is observed minus predicted stay; positive values indicate underprediction. CIs use 2000 bootstrap resamples. The median paired difference is calculated within patients and is not the difference between the two marginal medians.

## Data Availability

The data presented in this study are available on reasonable request from the corresponding author, subject to institutional and ethical restrictions related to patient confidentiality.

## Supplementary Materials

The following supporting information can be downloaded at: https://www.mdpi.com/article/10.3390/jcm15218387/s1. Supplementary Code S1 contains analysis.py, software requirements, a README, Supplementary Table S1 (outcome performance with analysis-status labels), Table S2 (calibration-group counts and estimates), Table S3 (variable and endpoint dictionary), aggregate results, and figures. No identifiable or patient-level clinical data are included.
